# Multidrug Resistance and Virulence Traits of *Salmonella enterica* Isolated from Cattle: Genotypic and Phenotypic Insights

**DOI:** 10.3390/antibiotics14070689

**Published:** 2025-07-08

**Authors:** Nada A. Fahmy, Sumin Karna, Angel Bhusal, Ajran Kabir, Erdal Erol, Yosra A. Helmy

**Affiliations:** 1 Department of Veterinary Science, Martin-Gatton College of Agriculture, Food, and Environment, University of Kentucky, Lexington, KY 40546, USA; 2 Veterinary Diagnostic Laboratory, Martin-Gatton College of Agriculture, Food, and Environment, University of Kentucky, Lexington, KY 40511, USA

**Keywords:** antimicrobial resistance, biofilm, MDR, motility, pathogenicity, *Salmonella*, virulence

## Abstract

**Background/Objective:** Non-typhoidal *Salmonella* is a leading cause of foodborne illness worldwide and presents a significant One Health concern due to zoonotic transmission. Although antibiotic therapy remains a standard approach for treating salmonellosis in severe cases in animals, the widespread misuse of antibiotics has contributed to the emergence of multidrug-resistant (MDR) *Salmonella* strains. This study provides insights into the genotypic and phenotypic characteristics among *Salmonella* isolates from necropsied cattle. **Methods:** A total of 1008 samples were collected from necropsied cattle. *Salmonella enterica* subspecies were identified by MALDI-TOF MS and subsequently confirmed by serotyping. The biofilm-forming ability of the isolated bacteria was assessed using a crystal violet assay. The motility of the isolates was assessed on soft agar plates. Additionally, the antimicrobial resistance genes (ARGs) and virulence genes were investigated. Antimicrobial resistance patterns were investigated against 19 antibiotics representing 9 different classes. **Results:** *Salmonella* species were isolated and identified in 27 necropsied cattle. *Salmonella* Dublin was the most prevalent serotype (29.6%). Additionally, all the isolates were biofilm producers at different levels of intensity, and 96.3% of the isolates exhibited both swarming and swimming motility. Furthermore, virulence genes, including *invA*, *hilA*, *fimA*, and *csgA*, were detected in all the isolates. The highest resistance was observed to macrolides (azithromycin and clindamycin) (100%), followed by imipenem (92.6%), and chloramphenicol (85.2%). All isolates were multidrug-resistant, with a multiple antibiotic resistance (MAR) index ranging between 0.32 and 0.74. The aminoglycoside resistance gene *aac(6′)-Ib* was detected in all the isolates (100%), whereas the distribution of other antimicrobial resistance genes (ARGs) varied among the isolates. **Conclusions:** The increasing prevalence of MDR *Salmonella* poses a significant public health risk. These resistant strains can reduce the effectiveness of standard treatments and elevate outbreak risks. Strengthening surveillance and regulating antibiotic use in livestock are essential to mitigating these threats.

## 1. Introduction

Livestock, such as cattle, sheep, and goats, serve as major reservoirs for diverse *Salmonella* serotypes and play a critical role in the transmission of infection to humans [1]. Nontyphoidal salmonellosis outbreaks are frequently associated with the consumption of contaminated meat, poultry, and processed food products [2]. According to the Centers for Disease Control and Prevention (CDC), 82.2% of Americans consume beef weekly, with 67% specifically consuming ground beef [3]. Consequently, nontyphoidal *Salmonella* is estimated to cause approximately 1.35 million infections annually, resulting in approximately 26,500 hospitalizations and 420 deaths in the USA. Thus *Salmonella* represents one of the highest economic burdens among foodborne pathogens, with total annual costs exceeding $17.1 billion [3,4,5]. Similarly, on a global scale, the World Health Organization (WHO) identifies *Salmonella* species among the 31 leading pathogens capable of causing both intestinal and systemic diseases in humans, contributing to an estimated 150 million illnesses and 60,000 fatalities annually worldwide [6,7,8]. However, the true burden of *Salmonella* infections is likely much higher, as the CDC estimates that only 1 in every 30 cases is laboratory confirmed, largely due to underdiagnosis and underreporting [9].

*Salmonella* species are antigenically classified according to the Kauffmann–White scheme, which differentiates over 2600 serotypes based on variations in somatic (O), flagellar (H), and capsular (K) antigens [10]. This significant diversity contributes to variation in host adaptation, pathogenic severity, and clinical outcomes [11]. *Salmonella enterica* subsp. *enterica* is the most prevalent subspecies and primarily infects warm-blooded animals [12]. The National Veterinary Services Laboratory has reported *Salmonella* Dublin as the most prevalent serotype in livestock, specifically cattle, followed by *Salmonella* Cerro and *Salmonella* Typhimurium [13]. While some serotypes are host-restricted, others can infect a wide range of animal species, contributing to their significance as zoonotic and foodborne pathogens [14]. The zoonotic transmission of *Salmonella* may occur through direct or indirect contact with infected animals or their environments, ingestion of contaminated animal-derived food or water, or via intermediate hosts, such as insect vectors [15,16]. Spillover to humans typically depends on the convergence of environmental conditions, pathogen characteristics, and host susceptibility [17,18]. In humans, high-risk groups, including infants, young children, the elderly, and immunocompromised patients, are particularly vulnerable, especially when hygiene is inadequate or when the gut microbiota is disrupted due to dysbiosis. In animals, the risk of infection is elevated by factors such as stress, co-infections, herd size, and suboptimal farm management practices [16,19].

The infection cycle of bovine salmonellosis typically begins with oral ingestion, especially in calves with immature gut microbiota or gastrointestinal stasis [20]. *Salmonella* adheres to and invades enterocytes and penetrates the intestinal lining, triggering inflammation or being engulfed by immune cells [21]. Once inside macrophages, it survives and disseminates through the lymphatic system, targeting lymphoid tissues and contributing to the development of bacteremia and systemic disease. Additionally, systemic spread can occur via pharyngeal lymphoid tissues, bypassing the intestinal tract [13,22]. Antibiotic therapy is frequently used as the initial strategy to manage infections caused by *Salmonella* spp. in both humans and animals. The extensive and often misuse of antibiotics in the livestock sector has raised significant concerns regarding the emergence and spread of antimicrobial-resistant genes (ARGs) [23]. Infections with multidrug-resistant (MDR) *Salmonella* are associated with a two-fold increase in the mortality risk compared to drug-susceptible strains [24]. The dissemination of MDR *Salmonella* has been closely linked to antibiotic misuse in food-producing animals, thereby facilitating zoonotic transmission. Approximately 70% of MDR *Salmonella* outbreaks in the U.S. have been attributed to livestock-derived foods [25]. In addition to the development of MDR, the *Salmonella* genome is encoded with a wide array of virulence factors that contribute to its pathogenicity and adaptability. *Salmonella* pathogenesis involves sequential steps critical for host colonization. The process begins with adhesion to intestinal epithelial cells via fimbriae and outer membrane proteins [26]. This is followed by invasion, mediated by *Salmonella* pathogenicity islands (SPIs) and a type 3 secretion system (T3SS), which injects effector proteins to disrupt host cell functions and facilitate bacterial entry. Upon entry into the host, *Salmonella* activates a range of virulence mechanisms, including the modulation of host signaling pathways and the inhibition of phagocytic killing to evade immune responses and establish a successful infection [26,27]. The differences in SPI gene content likely reflect the co-evolutionary dynamics and host-specific adaptations of SPIs across diverse *Salmonella* species and serotypes [28]. Similarly, virulence plasmids play a pivotal role in facilitating systemic dissemination following oral infection in animal models [29]. The pathogenicity of *Salmonella* reflects a sophisticated genomic architecture shaped by the evolutionary acquisition of pathogenicity islands and the horizontal transfer of genes and mobile genetic elements, equipping the organism with the metabolic flexibility, regulatory control, and host-specific virulence mechanisms required for successful intracellular survival and systemic infection [30,31,32,33].

Additional factors vital to *Salmonella* colonization include flagella-driven motility, fimbriae, and adhesins for epithelial attachment [34,35], as well as toxin production that disrupts host cell function [35,36]. A key component of its virulence strategy, *Salmonella* utilizes multiple secretion systems to translocate effector proteins that manipulate host processes and enhance intracellular survival. Four major systems have been identified: type 1, 3, 4, and 6 secretion systems (T1SS–T6SS), each contributing uniquely to infection and immune evasion [36]. In addition to these classical virulence factors, biofilm formation represents a critical survival strategy that significantly enhances *Salmonella* pathogenicity [37,38]. Bacterial cells growing within a biofilm adopt a markedly different mode of life compared to those in the planktonic state, with distinct patterns of gene and protein expression [39]. Within these structured communities, biofilms create protective microenvironments that support the development of both genotypic and phenotypic diversities. This dynamic nature enables bacteria to adapt rapidly to environmental stresses [40]. One of the hallmark features of biofilm-associated cells is their elevated resistance or tolerance to antimicrobial agents, which is not typically observed in their free-living cells [37].

While postharvest interventions target surface contamination, the pathogen can inhabit multiple internal organs, posing challenges for detection and control. Studies have identified *Salmonella* in the intestinal contents of clinically healthy cattle, as well as in liver and lung tissues, emphasizing the role of systemic dissemination and subclinical carriage [3]. Conventional culture methods and advanced molecular diagnostics, such as polymerase chain reaction (PCR), have been employed to assess *Salmonella* prevalence across various tissues [41,42]. These findings underscore the need for comprehensive preharvest surveillance strategies that extend beyond fecal sampling to assess infection status and reduce public health risks accurately [3,42]. In 2024, Kentucky’s livestock sector, led by beef production, played a key role in sustaining the state’s agriculture, with a 16.8% rise in receipts. As one of the top beef-producing states, this growth helped drive total agricultural cash receipts to nearly $8.3 billion [43]. Thus, in this study, we aimed to investigate the phenotypic and genotypic characteristics of *Salmonella* isolates from necropsied cattle in central Kentucky, with emphasis on antimicrobial resistance profiles, prevalent serotypes, biofilm formation ability, motility behavior, and the correlation between the phenotypic and the genetic traits.

## 2. Materials and Methods

### 2.1. Sample Collection, Bacterial Isolation, and Characterization

Between January 2022 and December 2023, a total of 1008 samples were collected from individual necropsied cattle submitted to the Veterinary Diagnostic Laboratory at the University of Kentucky. Sampling was conducted through random, case-based submissions from field veterinarians across various farms in central Kentucky, U.S. These cases were submitted and investigated at the request of the attending clinicians. Samples were collected as necropsied animals became available, and diagnostic evaluations were performed accordingly. These samples were collected from various organs, including the intestine, colon, lung, and liver. The animals’ ages ranged between 3 days and 12 years. They were categorized as follows: neonatal (2 to 28 days), calf (29 to 90 days), juvenile (3 months to 12 months), and adult (1 year and older) [44]. Organs were seared with sterile blades prior to sample collection to minimize surface contamination. Swab samples were then obtained and inoculated onto blood agar (BA), Hektoen enteric agar, and eosin methylene blue (EMB) agar (Hardy Diagnostics, Santa Maria, CA, USA). All primary cultures were incubated at 37 °C for 24 h. Swabs from intestinal samples were enriched in selenite broth at 37 °C for 24 h and then subcultured onto Hektoen and EMB agar, followed by incubation for an additional 48 h at 37 °C. Colonies exhibiting black pigmentation on Hektoen or a pale/white appearance on EMB were selected for identification by matrix-assisted laser desorption/ionization time-of-flight mass spectrometry (MALDI-TOF MS). Identification was performed using the direct transfer method, with a minimum score threshold of 1.7 for genus-level identification and 2.0 for species-level identification, as determined by the Biotyper software (version 4.0; Bruker Scientific Corp., San Jose, CA, USA). Serotyping was conducted at the National Veterinary Services Laboratory (NVSL) following the Kauffmann–White–Le Minor Scheme. The *Salmonella* serotype was determined based on the combination of O- and H-antigen profiles obtained from the agglutination results [45]. All isolates were preserved in 25% glycerol (*v*/*v*) and stored at −80 °C for further analysis.

### 2.2. Biofilm Formation Assay

The ability of the *Salmonella* isolates to form biofilm was evaluated using a crystal violet staining assay, as described previously [46,47]. An overnight culture of *Salmonella* was grown in Luria–Bertani (LB) broth (BD Difco™, Franklin Lakes, NJ, USA) and diluted to a final optical density (OD_600_) of ~0.05 (10^7^ CFU/mL). Aliquots of 100 μL of the bacterial suspensions were transferred into a 96-well microtiter plate for each isolate, with sterile LB media serving as a negative control. The plates were incubated statically at 37 °C for 48 h to allow biofilm formation. After incubation, planktonic cells were removed, and the wells were washed gently with distilled water to remove any unbound cells. To quantify biofilm formation, the remaining biofilms were stained with 160 μL of 0.1% crystal violet (*w*/*v*) for 15 min. The plates were then rinsed with distilled water to remove the extra stain and were allowed to dry for 60 min at room temperature. The crystal violet bound to the biofilms was solubilized using 30% acetic acid (*v*/*v*) per well. The biofilm formation was measured at OD_550_ nm using a microplate reader (Tecan, Morrisville, NC, USA). Each isolate was tested in eight replicates, and the assay was performed twice independently to confirm biological reproducibility. The data were presented as the mean absorbance ± standard deviation (SD). The *Salmonella* strains were considered biofilm producers when the OD_550_ value was at least three times the SD of the mean absorbance of the negative control (OD_NC_). The biofilm formation was categorized as a strong biofilm producer (SBP) (OD_550_ ≤ 4 OD_NC_), moderate biofilm producer (MBP) (2 OD_NC_ ≤ OD_550_ < 4 OD_NC_), weak biofilm producer (WBP) (OD_NC_ ≤ OD_550_ < 2 OD_NC_), and non-biofilm producer (OD_550_ ≤ OD_NC_), as previously described [48].

### 2.3. Motility Assay

Swarming and swimming motility were evaluated using soft agar plates, following the protocols described before [49,50]. The *Salmonella* isolates were cultured overnight in LB broth (BD Difco™, USA). For swimming motility, 6 μL of *Salmonella* culture (OD_600_~0.05) was inoculated into the center of a 0.25% soft agar plate supplemented with 0.5% glucose. For the swarming motility, 6 μL of the same *Salmonella* culture (OD_600_~0.05) was spotted onto the center of a 0.5% soft agar plate, supplemented with 0.5% glucose. The plates were incubated at 37 °C, and the formation of a turbid zone was recorded at 6 h for swimming motility. In contrast, the swarming zone was measured at 12 h to evaluate the bacteria’s movement on soft agar. *Proteus mirabilis* (*P. mirabilis*) ATCC 35659 and *S*. Typhimurium ATCC 14028 were used as positive controls, while *Rhodococcus equi* was included as a negative control. The isolates were considered motile when a diffuse growth zone expanded outward from the inoculation or spotting area and negative if the growth remained confined to the inoculation point [51,52]. Three independent replicates were performed for each experiment, and the assay was performed twice.

### 2.4. Antimicrobial Susceptibility Testing (AST)

The antimicrobial susceptibility profiles of the *Salmonella* isolates were determined using the broth microdilution method (Thermo Scientific™ Sensititre™ Vet Bovine BOPO7F Plate, Waltham, MA, USA) that contain antibiotics, including neomycin (NEO), gentamicin (GEN), ampicillin (AMP), ceftiofur (CEF), tetracycline (TET), enrofloxacin (ENR), florfenicol (FLO), and clindamycin (CLI). Additional antibiotics were not included in the panel: amikacin (AMK), amoxicillin–clavulanic acid (AMC), meropenem (MEM), imipenem (IPM), ceftazidime (CAZ), doxycycline (DOX), trimethoprim–sulfamethoxazole (SXT), levofloxacin (LVX), ciprofloxacin (CIP), chloramphenicol (CHL), and azithromycin (AZM). The concentrations used for each antibiotic, along with their abbreviations and corresponding references, are detailed in Table S1. All the *Salmonella* isolates were cultured on Columbia agar plates supplemented with 5% sheep blood (Becton Dickinson, Franklin Lakes, NJ, USA) and incubated at 37 °C for 24 h. Antimicrobial susceptibility testing (AST) was conducted, following the manufacturer’s protocol. In brief, a 0.5 McFarland standard suspension was prepared in distilled water using a spectrophotometer. From this suspension, 10 µL was added to Sensititre Mueller Hinton broth. Subsequently, 50 µL of the prepared mixture was dispensed into each well of the Sensititre™ Vet Bovine BOPO7F Plate. The plate was incubated at 37 °C, and MICs were determined after 18 h using the Thermo Scientific Sensititre OptiRead Automated Fluorometric Plate Reading System. For the additional antibiotics not included in the commercial panel, AST was performed using a standard broth microdilution method. A two-fold serial dilution of the antibiotic was prepared in a sterile 96-well microtiter plate, and 50 µL of the bacterial suspension (prepared as above) was added to each well. The plates were incubated at 37 °C for 18 h, and MICs were determined using the same OptiRead™ system. The interpretive breakpoints for each antimicrobial agent were established based on CLSI M100 and CLSI VET01S guidelines [53,54]. The multiple antibiotic resistance (MAR) index was calculated as the ratio of the number of antibiotics (19) to which the strain is resistant to the total number of antibiotics tested [55]. The MAR index exceeding 0.2 typically indicates that the isolates originate from high-risk sources with frequent antibiotic exposure [56,57]. Strains were considered MDR if they were non-susceptible to at least one agent in three or more antimicrobial classes. In contrast, strains were considered as extensively drug-resistant (XDR) if they were resistant to at least one agent in all but one or two antimicrobial categories, remaining susceptible to only one or two classes of antibiotics [57,58].

### 2.5. Detection of Virulence Genes and Antimicrobial Resistance Genes (ARGs)

All the *Salmonella* isolates collected in this study were screened for the presence of virulence genes using conventional PCR. Genomic DNA was extracted from overnight cultures of the *Salmonella* isolates using a boiling method, as previously described [42,59,60]. Briefly, a single colony of each *Salmonella* isolate was suspended in 100 µL of molecular-grade water and subjected to heat lysis using a thermal cycler at 95 °C for 10 min, followed by immediate chilling on ice for 10 min. The lysate was then centrifuged at 5000× *g* for 10 min, and 50 µL of the supernatant was collected as the DNA template and stored at −20 °C. DNA quality and quantity were assessed using a Thermo Scientific NanoDrop spectrophotometer (Thermo Fisher, Lexington, KY, USA). Seventeen virulence genes were selected, based on their specificity to pathogenic *Salmonella* isolates, conservation across species, and functional relevance to host invasion and survival. These genes included host cell invasion and intracellular survival genes (*invA*, *hilA*, *intA*, *intB*), adhesion and attachment genes (*fimA*, *fimD*), a type 3 secretion system (T3SS) (*spiA*, *spi4D*), a type 1 secretion system (T1SS) (*siiA*, *siiC*), host immune suppressor genes (*spvC, sopB*), biofilm formation genes (*csgA*, *csgB*), and motility and flagellar genes (*fljB*, *fliC*, *flhD*). Likewise, fifteen ARGs, representing different antibiotic classes, were selected and screened for their presence among the *Salmonella* isolates. These included genes encoding antimicrobial resistance to β-lactamase (*bla_TEM_*_-1B_, *bla_CMY_*, *bla_CTX-M_*, *bla_SHV_*, *bla_OXA-9_*), aminoglycoside (*aadB*, *aacA*3, *aac(6′*)), tetracycline *(tetB),* phenicol (*floR*, *catB*), sulfonamide (*sul2*), macrolides (*ermB2*), streptomycin (*str*), and colistin (*mcr1-9*). The primers for each targeted virulence and antimicrobial resistance gene are listed in Tables S2 and S3.

PCR reactions were performed in a total volume of 25 μL, containing 12.5 μL of 2X GoTaq Green Master Mix (Promega, Madison, WI, USA), 0.5 μL of each forward and reverse primer (stock solution 10 μm), 2 μL of DNA template, and nuclease-free water adjusted to the final volume of 25 μL. The thermal cycling conditions were optimized for each primer set, typically involving an initial denaturation step at 94 °C for 5 min, followed by 30–35 cycles of denaturation at 94 °C for 30 s, annealing at the appropriate temperature for 30 s, and extension at 72 °C for 1 min, with a final extension step at 72 °C for 7 min. The PCR amplicons were separated using agarose gel electrophoresis with 1.5% agarose gels stained with ethidium bromide, and then visualized using a gel documentation system (Bio-Rad, Hercules, CA, USA).

### 2.6. Statistical Analysis

A two-way Analysis of Variance (ANOVA) was employed to evaluate the effects of two independent variables on the categorical outcomes of the biofilm development and motility experiments. The relationship between the sample type and *Salmonella* prevalence was evaluated using Fisher’s Exact Test, and descriptive statistics were used to calculate the 95% confidence interval (CI). This method is preferred over the normal approximation, especially for small sample sizes or proportions near 0 or 1. These analyses were conducted using the R Studio software (2025.05.0+496) [61]. The hierarchical clustering heatmaps were generated using the Complex Heatmap package in RStudio [62]. The Pearson’s correlation coefficient was calculated to assess the relationship between the ARGs and virulence genes. The correlation matrix was illustrated using the R Studio Corr package, providing a clear graphical representation of the relationships among the variables [63]. The statistical significance was determined at a *p*-value of less than 0.05 for all analyses.

## 3. Results

### 3.1. Occurrence and Serotype Distribution of Salmonella

Out of 1008 necropsied cattle, 27 (2.7%) were positive for *Salmonella* isolates, with a prevalence of 59.3% (*n* = 16) in females, compared to 40.7% (*n* = 11) in males. Among the positive cases, the highest occurrence was observed in adult cattle (≥1 year), accounting for 37.0% (10/27; 95% CI: 18.8–55.3), followed by juveniles (3–12 months), with a prevalence of 33.3% (9/27; 95% CI: 15.6–51.1). Calves (29–90 days) represented 18.5% (5/27; 95% CI: 3.9–33.2), while neonates (2–28 days) had the lowest occurrence at 11.1% (3/27; 95% CI: 0.0–23.0). Most of the isolates, 77.8% (21/27), were recovered from the intestine (95% CI: 62.1–93.5). Conversely, the lowest occurrence (3.7%) was from the lungs and kidneys (1/27; 95% CI: 0.0–10.8). Notably, *S*. Dublin was the most prevalent serotype within the isolates (29.6%; 8/27; 95% CI: 12.4–46.9). This was followed by *S.* Typhimurium, *S.* Muenster (11.1% each), *S.* Thompson, *S.* Worthington, *S.* Montevideo, and *S.* Anatum (7.4%). Additionally, *S.* Cerro, *S.* Hartford, *S.* Meleagridis, *S.* Newport, and *S.* III 38:(k): z35 were detected in 3.7% of the isolates (Table 1 and Table S4).

### 3.2. Biofilm Formation Within the Salmonella Isolates

**Figure 1:**
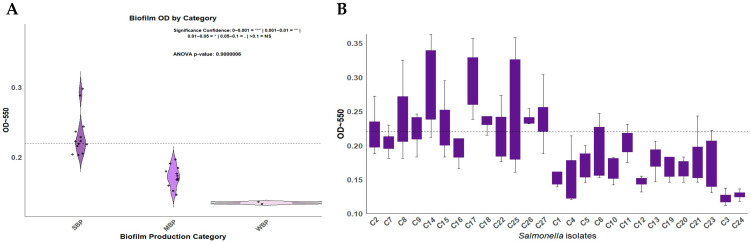
The quantification and categorization of biofilm formation among the bacterial isolates. (**A**) A box plot illustrating the mean biofilm biomass of the isolated bacteria using the crystal violet assay. The y-axis represents optical density (OD_550_), and the x-axis lists the corresponding bacterial isolates. The error bars represent the standard deviation across the replicates. (**B**) A violin plot illustrating biofilm formation within the *Salmonella* isolates (OD_550_). The samples were categorized as strong biofilm producers (SBPs), moderate biofilm producers (MBPs), and weak biofilm producers (WBPs). A horizontal dashed line represents a cutoff value.

The biofilm production ability of the *Salmonella* isolates was evaluated, and varying levels of biofilm formation were observed among the tested isolates. The results showed that 48.2% (13/27) of the isolates were SBPs (95% CI: 29.30–66.99%), while 44.4% (12/27) were MBPs (95% CI: 25.70–63.19%), and 7.4% (2/27) were WBPs (95% CI: 0.00–17.29%) (Figure 1A). The mean optical density (OD_550_) values indicated biofilm biomass ranges between 0.1 and 0.3 (Table S5). Isolates C14, C19, and C25 showed the highest OD_550,_ while C3 and C24 possessed the lowest OD_550_ (Figure 1B).

### 3.3. Swimming and Swarming Motility

**Figure 2:**
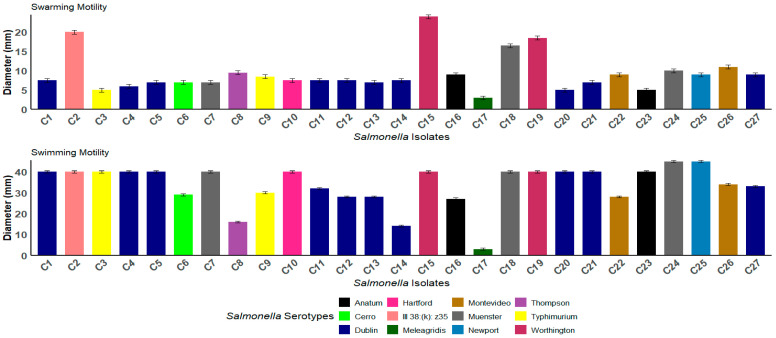
The swarming and swimming motility of the *Salmonella* isolates. The bar graphs represent the average motility diameters (mm) for each isolate. Each bar corresponds to a distinct *Salmonella* isolate, and the color coding indicates the different serotypes. The swarming and swimming motility assays were conducted on semi-solid agar, and the measurements were recorded after incubation. The error bars represent the standard deviations.

The motility of the *Salmonella* strains was assessed for surface swarming (growth on media with 0.5% agar) and swimming (growth on media with 0.25% agar). Out of the 27 isolates, 96.3% (26 out of 27) exhibited swimming, displaying either a featureless or bull’s-eye morphology, with an average of 33.8 ± 0.2 mm. Additionally, 96.3% of strains demonstrated the ability to swarm along the agar surface, forming colonies that were featureless, smooth, and flat. The featureless colonies resulted from the cells spreading evenly and continuously outward from the inoculation point as a monolayer, with an average of 29.3 ± 0.2 mm (Figure S1). The highest swarming motility was observed in C2 (*S.* III 38:(k): z35) and isolates C15 and C19, which are *S.* Worthington (Figure 2). A chi-square test of independence was conducted. The results indicated no statistically significant association between the serotype and swarming motility (χ^2^ = X, *p* = 0.134), nor between the serotype and swimming motility (χ^2^ = Y, *p* = 0.349).

### 3.4. Distribution of Virulence Genes Among Salmonella Isolates

**Figure 3:**
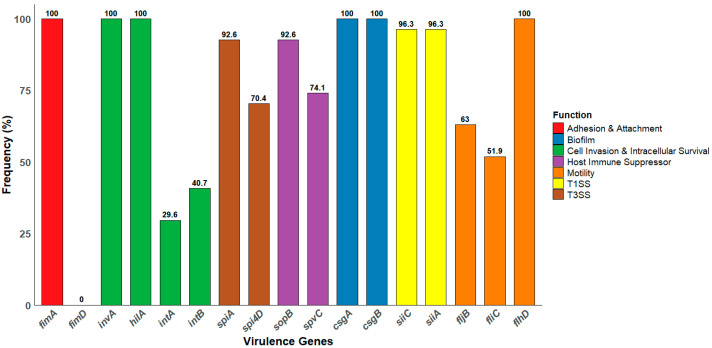
The frequency distribution of the virulence genes among the *Salmonella* isolates. The bar chart illustrates the percentage of isolates harboring each gene. The data reflect the prevalence of specific genes associated with pathogenicity, including those involved in adhesion and attachment, biofilm formation, host invasion, motility, a type 1 secretion system (T1SS), and a type 3 secretion system (T3SS).

Molecular screening for virulence genes revealed that all the isolates (100%, *n* = 27) harbored *invA*, *hilA*, *fimA*, *csgA*, *csgB*, and *flhD*, indicating a consistent trait of invasion, motility, and biofilm formation. Additionally, 96.3% (26/27) of the isolates carried both *siiA* and *siiC* genes, which are components of TISS, while 92.6% (25/27) of the isolates harbored the *spiA* gene, a key effector within SPI-2. Moreover, genes involved in host immune modulation, such as *sopB* and *spvC*, were detected in 92.6% (25/27) and 74.1% (20/27) of the isolates, respectively. Interestingly, all isolates harbored at least one of the motility-associated genes *fljB*, *fliC*, or *flhD*, confirming their genetic capacity for flagellar-driven motility. The gene-specific prevalence is illustrated in Figure 3.

### 3.5. Antibiotic Susceptibility Profile

**Figure 4:**
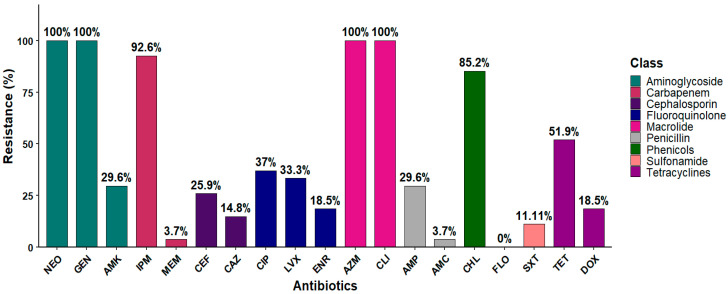
The antibiotic resistance frequency within the collected *Salmonella* isolates. The bar chart illustrates the percentage of isolates resistant to antibiotics within each antimicrobial class. All the isolates possessed 100% resistance to NEO, GEN, CLI, and AZM. Neomycin (NEO), gentamicin (GEN), amikacin (AMK), amoxicillin and clavulanic acid (AMC), ampicillin (AMP), meropenem (MEM), imipenem (IPM), ceftazidime (CAZ), ceftiofur (CEF), tetracycline (TET), doxycycline (DOX), trimethoprim and sulfamethoxazole (SXT), levofloxacin (LVX), ciprofloxacin (CIP), enrofloxacin (ENR), florfenicol (FLO), chloramphenicol (CHL), azithromycin (AZM), and clindamycin (CLI).

**Figure 5:**
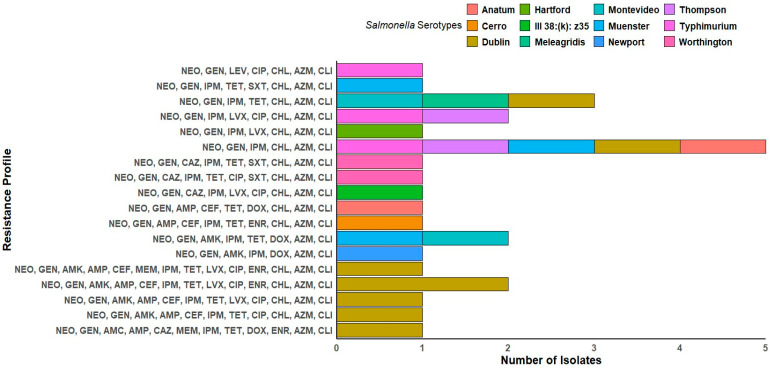
The distribution of antimicrobial resistance profiles among the *Salmonella* isolates, categorized by serotype. Each row represents the number of isolates sharing the same resistance profile, with the colors indicating the different *Salmonella* serotypes.

The antibiotic susceptibility testing for the *Salmonella* isolates was performed using broth microdilution against 19 antibiotics that cover nine different antimicrobial classes. Our results showed that 92.6% (25 out of 27) of the isolates were resistant to carbapenem (imipenem), and 85.2% (23 out of 27) were resistant to chloramphenicol. Similarly, 51.9% of the isolates (14 out of 27) were resistant to tetracycline, and 18.5% (5 out of 27) to doxycycline. In contrast, meropenem demonstrated the highest susceptibility, with activity observed against most isolates, 96.3% (26 out of 27), followed by trimethoprim–sulfamethoxazole (88.9%; 24 out of 27) (Figure 4 and Table S6). A total of 18 distinct phenotypic antimicrobial resistance profiles were identified among the 27 *Salmonella* isolates. The most prevalent profile, observed in five isolates across various serotypes, including *S.* Dublin, *S.* Muenster, *S.* Anatum, *S.* Thompson, and *S.* Typhimurium, included resistance to NEO, GEN, IPM, CHL, AZM, and CLI. Notably, *S.* Dublin exhibited the highest diversity of resistance profiles, being associated with eight distinct profiles. Multi-drug resistance (MDR) patterns frequently involved aminoglycosides (NEO and GEN), β-lactams (AMP, CEF, and CAZ), tetracyclines (TET and DOX), phenicols (CHL), fluoroquinolones (LVX, CIP, and ENR), macrolides (AZM), and lincosamides (CLI). One isolate of *S.* Montevideo and one of *S.* Muenster shared an identical profile (NEO, GEN, AMK, IPM, TET, DOX, AZM, and CLI). In contrast, some profiles were serotype-specific, such as the extended resistance profile found only in *S.* Dublin (NEO, GEN, AMK, AMP, CEF, MEM, IPM, TET, LVX, CIP, ENR, CHL, AZM, and CLI) (Figure 5). All the isolates were classified as MDR, as each demonstrated resistance to at least one antibiotic in three or more antimicrobial classes. Interestingly, 100% (*n* = 27) of the isolates were MDR to macrolides (azithromycin and clindamycin). Likely, 100% (*n* = 27) of the isolates were MDR to aminoglycoside (neomycin), and 29.6% (*n* = 8) to aminoglycoside (neomycin, gentamicin, and amikacin). The MAR index was calculated for each isolate, with values ranging from 0.32 to 0.74, indicating MDR across all the isolates. Notably, strain C14 exhibited the highest MAR index at 0.74, followed by C11 at equal to 0.68, and C5 and C12, with a MAR index equal to 0.63. These isolates are considered XDR as they were susceptible to only one or two classes of antibiotics (Table S7).

### 3.6. Distribution of ARGs Among Salmonella Isolates

**Figure 6:**
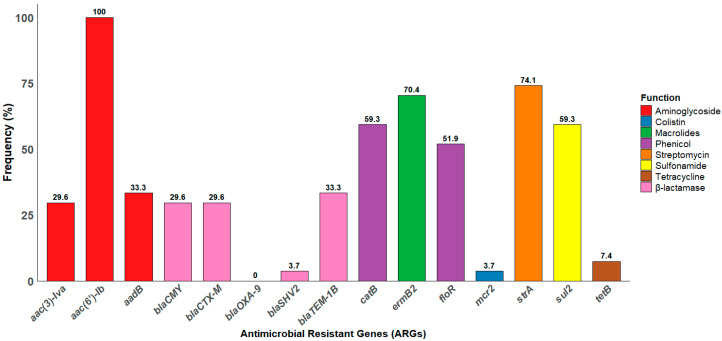
The frequency distribution of antimicrobial-resistant genes among the *Salmonella* isolates. The bar chart illustrates the percentage of isolates harboring each gene. All the isolates harbor aminoglycoside resistance genes *(aac(6′)-Ib)*.

Our *Salmonella* isolates harbored a variety of antimicrobial resistance genes (ARGs), including the aminoglycoside resistance gene *aac(6′)-Ib*, which was detected in all the isolates (100%; 27/27). The prevalence of other ARGs varied across the isolates; notably, the streptomycin resistance gene (*strA*) was detected in 74.1% (20/27) of the isolates, while macrolides (*ermB2*) were detected in 70.1% (19/27). Similarly, phenicol resistance genes (*floR* and *catB*) were identified in (59.3%, *n* = 16) and (51.9%, *n* = 14), respectively. Additionally, 59.3% (16/27) of the isolates harbor the sulfonamide resistance (*sul2*) gene. Interestingly, the β-lactamase resistant gene (*bla_OXA-9_*) was not detected in any of the isolates. Among the screened *mcr*-1 to *mcr*-9 genes, only *mcr*-2 was detected in the *Salmonella* isolates, with a prevalence of 3.7% (1 out of 27). The frequency of each gene is illustrated in Figure 6.

### 3.7. Phenotypic and Genotyping Correlation

**Figure 7:**
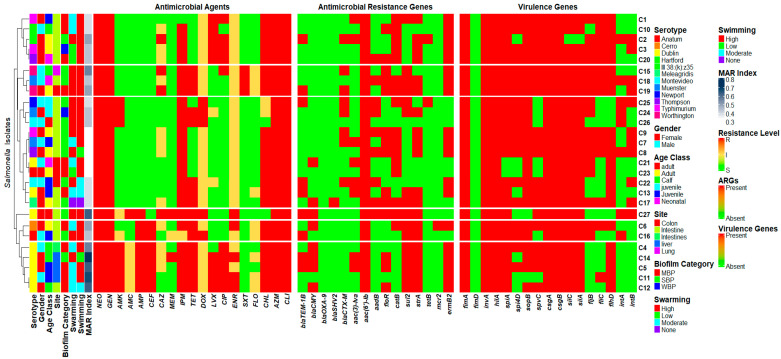
Hierarchical clustering was performed based on the phenotypic resistance profiles of the *Salmonella* isolates, resulting in six distinct clades, visualized as row splits with gaps. The same dendrogram structure was applied across the heatmaps, representing the presence of virulence genes, antimicrobial resistance gene (ARG) profiles, and phenotypic profiles. Phenotypic data include the MAR index, swimming, swarming, and biofilm category, age class, gender, serotype, and site of isolation for each isolate. Neomycin (NEO), gentamicin (GEN), amikacin (AMK), amoxicillin and clavulanic acid (AMC), ampicillin (AMP), meropenem (MEM), imipenem (IPM), ceftazidime (CAZ), ceftiofur (CEF), tetracycline (TET), doxycycline (DOX), trimethoprim and sulfamethoxazole (SXT), levofloxacin (LVX), ciprofloxacin (CIP), enrofloxacin (ENR), florfenicol (FLO), chloramphenicol (CHL), azithromycin (AZM), and clindamycin (CLI).

For the phenotypic data, hierarchical clustering of the *Salmonella* isolates was performed based on their phenotypic antibiotic resistance profiles. The resulting dendrogram, derived from the phenotypic resistance matrix, was used to align all virulence genes, ARGs, and phenotypic profiles. Importantly, isolates C4, C5, C11, C12, and C14 were clustered into distinct clades and classified as XDR, demonstrating resistance to multiple antibiotic classes. These isolates were all recovered from calf and juvenile animals and belonged exclusively to the *S*. Dublin serotype. Interestingly, most of the *S.* Dublin isolates demonstrated exceptionally high MAR index values (>0.5). Furthermore, all the isolates in this sub-clade carried the macrolide resistance gene (*ermB2*), sulfonamide resistance gene (*sul2*), and the streptomycin resistance gene (*strA*). Conversely, isolates C1, C2, C3, C10, and C20 were also clustered together and demonstrated lower resistance levels. Notably, all the isolates in this case were encoded by the phenicol-resistant gene (*catB*). These isolates were recovered from juvenile and calf cattle, specifically from intestinal sites, and demonstrated high swimming motility. Interestingly, despite their low resistance profiles, these isolates harbored a broad array of virulence genes. In parallel, an analysis of virulence gene distribution revealed that isolates *S.* Thompson (C8) and *S.* Muenster (C18) harbored the most virulence-associated genes (94.1%, *n* = 16) and clustered within a distinct subclade. Both were recovered from the intestine and exhibited high swarming motility and strong biofilm-forming ability. In contrast, isolates C21 (*S.* Dublin) and C23 (*S.* Anatum) possessed fewer virulence genes and clustered separately. Notably, both isolates were recovered from the colon and exhibited moderate biofilm-forming ability Figure 7.

**Figure 8:**
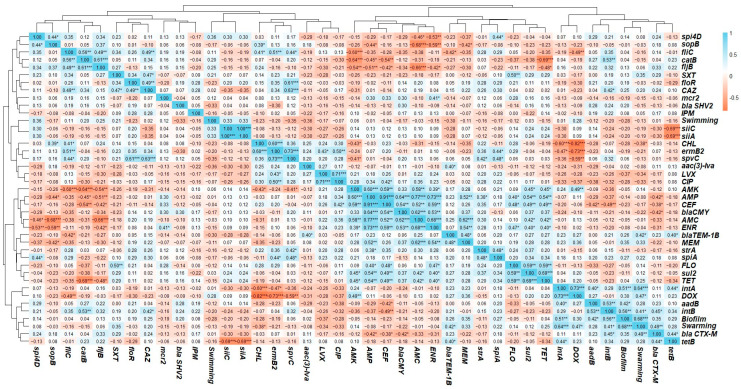
Correlation heatmap with a hierarchical clustering correlation coefficient (*r*) matrix between the phenotypic and genotypic patterns. The asterisks next to the correlation coefficients indicate statistical significance; *** (*p* < 0.001), ** (*p* < 0.01), * (*p* < 0.05), and no asterisk indicates *p* ≥ 0.05 (not significant). Neomycin (NEO), gentamicin (GEN), amikacin (AMK), amoxicillin and clavulanic acid (AMC), ampicillin (AMP), meropenem (MEM), imipenem (IPM), ceftazidime (CAZ), ceftiofur (CEF), tetracycline (TET), doxycycline (DOX), trimethoprim and sulfamethoxazole (SXT), levofloxacin (LVX), ciprofloxacin (CIP), enrofloxacin (ENR), florfenicol (FLO), chloramphenicol (CHL), azithromycin (AZM), and clindamycin (CLI).

Pearson’s correlation coefficients were calculated to assess the relationships between phenotypic and genotypic profiles, with significance levels evaluated to confirm the strength of these associations. Notably, the *intA* gene (*Salmonella* integron A) exhibited a significant positive correlation with doxycycline resistance (*r* = 0.73; *p* < 0.001) and with the *tetB* gene (tetracycline resistance) (*r* = 0.44; *p* < 0.05). Additionally, *intA* was positively correlated with the *aadB* gene (*r* = 0.40; *p* < 0.05). Similarly, *intB* (*Salmonella* integron B) demonstrated a positive correlation with both *aadB* (aminoglycoside resistance gene) and *catB* (phenicol resistance gene) (*r* = 0.53; *p* < 0.01), as well as with *bla_CTX-M_* (β-lactam resistance gene) (*r* = 0.44; *p* < 0.05). Furthermore, significant correlations were also observed among the virulence genes. Positive correlations were observed between genes encoding components of the T3SS and host immune modulators. Specifically, *spi4D* correlated positively with both *sopB* and *spiA* (*r* = 0.44; *p* < 0.05), and *spiA* showed a significant positive correlation with *spvC* (*r* = 0.48; *p* < 0.05). Importantly, *siiA* and *siiC*, components of the T1SS, exhibited a perfect correlation (*r* = 1.00; *p* < 0.001). Additionally, correlation analysis revealed that biofilm production was positively associated with swarming motility (*r* = 0.68; *p* < 0.001), whereas a non-significant correlation was observed with swimming motility (*r* = −0.30). Additionally, a significant positive correlation was observed between the *bla_TEM_* (β-lactam resistance gene) and resistance to both meropenem (*r* = 0.48; *p* < 0.05) and ampicillin (*r* = 0.25). Similarly, the *bla_CMY_* (β-lactam resistance gene) showed significant positive correlations with resistance to amoxicillin–clavulanic acid (*r* = 0.62; *p* < 0.001), ampicillin (*r* = 0.64; *p* < 0.001), and ceftiofur (*r* = 0.54; *p* < 0.001). The correlation analysis of antibiotic resistance profiles revealed several significant associations, with strong positive correlations observed particularly among resistance to related antibiotic classes, such as β-lactams. For instance, resistance to ampicillin was positively correlated with resistance to amoxicillin–clavulanic acid (*r* = 0.58; *p* < 0.001) and ceftiofur (*r* = 0.59; *p* < 0.001). Moderate to strong correlations were also observed between resistance to meropenem and other β-lactam antibiotics like amoxicillin–clavulanic acid (*r* = 0.62; *p* < 0.001) and ampicillin (*r* = 0.52; *p* < 0.001), indicating possible cross-resistance or overlapping resistance pathways (Figure 8).

## 4. Discussion

*Salmonella* is recognized as one of the most prominent foodborne pathogens that is responsible for the highest economic losses globally [64]. The prevalence of *Salmonella* in cattle is a major public health concern due to the risk of zoonotic transmission and contamination through the food chain [65]. This study investigates the presence of *Salmonella* in necropsied cattle submitted to a veterinary diagnostic laboratory. It also investigates their antimicrobial resistance profiles and key virulence factors, including biofilm formation, motility, and the presence of ARGs and virulence-associated genes.

In our study, *Salmonella* was detected in 2.7% of 1008 necropsied cattle collected from January 2022 to December 2023. These findings align with earlier surveillance conducted between 2008 and 2019, during which *Salmonella* was detected in 2.5% of necropsied cattle from dairy farms across different states [66]. While this prevalence is lower than the pooled global estimate of 9% (95% CI: 7–11%) from 71 publications and 75 datasets, it falls within the range of global prevalence variations observed across continents. For instance, the pooled analysis shows that *Salmonella* prevalence in healthy cattle ranged from as low as 2% in Europe to as high as 16% in North America [67]. A Similar prevalence appears consistent across multiple animal populations, with previous reports of 1.2% in necropsied horses [45] and 1.4% among wildlife species (birds, mammals, and reptiles) [68]. In companion animals, a U.S. survey of 2965 veterinary clinical samples (2012–2014) found *Salmonella* prevalence rates of <1% in cats and 2.5% in dogs, with raw food representing a significant risk factor [69]. Recent surveillance of 2710 dairy cattle samples in Italy reported a 4.43% *Salmonella* prevalence, which is slightly higher than rates reported in other animal populations [70]. The variation in prevalence may be attributed to differences in isolation sites, as environmental and regional factors can significantly influence the presence of *Salmonella* [67]. Higher prevalence rates have often been associated with specific sampling sites, such as fecal and rectal samples [71]. In the present study, 21 out of 27 isolates (77.8%) were recovered from intestinal tissues, indicating it as the predominant site of isolation (95% CI: 62.1–93.5, *p* < 0.0001) (Table 1). *Salmonella* infection depends on the pathogen’s ability to outcompete the gut microbiota and induce inflammation, creating a niche for colonization [72]. Consistent with previous studies, the intestine was identified as the primary site of *Salmonella* isolation in both humans and animals, including cattle [67,73,74]. Although younger calves are often considered more susceptible to *Salmonella* due to their immature immune systems, limited gut microbiota, and higher susceptibility to co-infections, our study observed higher prevalence in juveniles (33.3%) and adults (37.0%). In juveniles, the dietary transition during weaning from milk to solid feed can disrupt gut microbiota stability, potentially facilitating *Salmonella* colonization [75,76]. The lower prevalence observed in calves (18.5%) and neonates (11.1%) may be partially explained by limited environmental exposure and the protective effect of passive immunity acquired through colostrum intake during the early postnatal period [77]. Additionally, prior antimicrobial treatments commonly administered during early growth stages may alter intestinal microbial balance and select for *Salmonella* colonization, including MDR strains [76].

Serotype-specific variations in the O-antigen structure enhance immune evasion and persistence, favoring survival in the nutrient-rich, permissive intestinal environment [78,79]. The most commonly reported *Salmonella enterica* serovars in cattle are *S.* Dublin, *S.* Typhimurium, *S.* Newport, *S.* Cerro, and *S.* Montevideo, with *S.* Dublin being host-adapted and frequently associated with systemic disease [67]. Among the *Salmonella* isolates recovered in this study, *S*. Dublin was the most abundant serotype, accounting for 29.6% (8/27, 95% CI: 12.4–46.9, *p* < 0.05) (Table 1), which is consistent with previous findings where *S.* Dublin was also the most frequently recovered serotype from bovines [80,81]. Additionally, *S.* Typhimurium was the second most commonly identified serotype in this study, consistent with global reports, underscoring its importance in bovine infections [82]. A recent meta-analysis estimated its prevalence at 10.1% among *Salmonella* isolates from cattle, establishing it as a key serotype in bovine salmonellosis [67]. In the National Animal Health Monitoring System (NAHMS), *S.* Typhimurium was also identified among *Salmonella* serotypes in U.S. beef cow–calf operations, though at a relatively low prevalence of 7.7% [83]. While *S.* Cerro, *S.* Newport, and *S.* Muenster are commonly reported in cattle at lower prevalence rates, this finding aligns with the distribution observed in this study [13,67]. Although earlier reports primarily highlighted *S.* Typhimurium and *S.* Dublin as the predominant serotypes isolated from cattle and beef products, more recent surveillance, including the present study, reveals a broader diversity of *Salmonella* serotypes circulating in cattle [13,16,84,85].

*Salmonella* spp. can produce biofilm, enhancing their survival and pathogenicity. Bacteria within biofilms exhibit increased resistance to antimicrobials, physical stresses, and host immune responses [38]. All *Salmonella* isolates exhibited biofilm production at varying levels, with 48.2% classified as strong biofilm producers. The *p*-value of 0.000264 is very small, indicating that the difference between at least two of the biofilm categories —SBPs, MBPs, and WBPs is statistically significant (*p* < 0.01). Notably, all the isolates harbor the curli amyloid fiber genes *csgA* and *csgB* (Figure 2), aligning with their observed biofilm-forming phenotypes. These findings are consistent with previous research demonstrating that the deletion of *csgA* impairs biofilm formation in *Salmonella* spp. [86,87,88]. The presence of these genes across all isolates suggests a conserved mechanism among *Salmonella* strains for establishing biofilms, which may contribute to their resilience in diverse environments and hosts [89]. Beyond biofilm formation, motility is a critical factor in *Salmonella* pathogenesis, facilitating both colonization and host invasion [90]. The widespread ability of bacteria to swarm on such media suggests their importance in surface colonization in natural environments. It is plausible that the gelatinous surfaces of animal tissues can mimic agar-like properties [91]. In our study, all isolates exhibited motility, demonstrating both swimming and swarming behavior on agar media of different concentrations, except for the non-motile *S.* Meleagridis (C17) (Figure S1). Despite harboring the flagellar genes *fliC*, *fljB*, and *flhD,* the isolate exhibited a non-motile phenotype. This discrepancy between genotype and phenotype may result from multiple factors, including gene regulation, point mutations, and epigenetic silencing [92,93]. Specifically, the presence of prophage elements that interfere with flagellar expression or assembly, as has been reported in *E. coli K-12*. The prophage regulator AppY enhances stress tolerance and biofilm formation while suppressing motility by downregulating *FlhDC*. A similar mechanism may explain the non-motile phenotype in *S.* Meleagridis (C17), despite intact flagellar genes, suggesting that prophage elements may modulate motility-related gene regulation [94,95,96].

Among the isolates, *S.* Worthington (C15 and C19) demonstrated the highest swarming and swimming motility. However, recent findings suggest that host factors can modulate bacterial motility. Beyond this, certain strains in our study exhibited high swarming motility but low swimming motility; this observation is in line with previous findings suggesting that bacterial strains can display diverse motility patterns influenced by environmental conditions and genetic regulation [91]. Such variability in motility may contribute to differences in biofilm architecture, consistent with the model proposed by Shrout et al., where early swarming behavior impacts the final structure of the biofilm through coordinated regulation of motility and matrix production [97,98]. Of note, the negative correlation between swimming and swarming motilities (*r* = −0.18) was not statistically significant, indicating that these behaviors are largely independent. In contrast, swarming motility, which involves coordinated surface movement, showed a significant positive correlation with biofilm formation (*r* = 0.68, *p* < 0.001) (Figure 8). In contrast, swimming motility, representing individual movement in liquid environments, appeared to play a lesser role in biofilm development. These findings support that surface-associated motility modes, rather than planktonic motility, are more closely linked to biofilm architecture and persistence [51,91,99].

Alongside biofilm formation and motility, the pathogenic success of *Salmonella* is fundamentally dependent on its ability to invade host cells, evade immune responses, and establish colonization. Cellular invasion is primarily mediated by the T3SS encoded within *Salmonella* SPI-1, which injects effector proteins into host epithelial cells [100]. All the *Salmonella* isolates in our study harbor adhesion and invasion genes (*fimA, hilA,* and *invA*), reflecting their conservation in association with adhesion and invasion mechanisms [101]. Additionally, they encode a broad array of effector proteins and host immune suppressors, reflecting their potential to modulate host cell signaling, evade immune detection, and enhance intracellular survival [102]. *Salmonella* is known as the *Salmonella*-containing vacuole (SCV), where it utilizes a second T3SS encoded by SPI-2 to manipulate host trafficking pathways, avoid lysosomal degradation, and promote intracellular replication [103,104]. Through the activation of SPI-1, *hilA* induces expression of effector proteins, which manipulate host cytoskeletal dynamics, promoting bacterial uptake via membrane ruffling [105]. Furthermore, 74.1% of our isolates are encoded by *spvC,* which is essential for systemic infection and functions as a phosphor–threonine lyase that disrupts host MAPK signaling, aiding in immune evasion [106]. The widespread presence of virulence factors highlights the isolates’ strong potential for invasion and immune evasion.

Due to growing concerns about antibiotic resistance, we analyzed antimicrobial resistance patterns and MAR indices to assess the potential for MDR and its implications for public health. All the isolates have a MAR index exceeding 0.2, a threshold that is widely recognized as indicative of high-risk sources where antibiotics are frequently used [55]. Moreover, all the isolates were classified as multidrug-resistant (MDR), as they were non-susceptible to antibiotics from more than three different classes [58]. Among our isolates, we observed the highest susceptibility to meropenem (96.3%) and trimethoprim–sulfamethoxazole (88.9%). These drugs are *strictly restricted* in veterinary practice. Conversely, we found higher resistance to aminoglycosides (neomycin and gentamycin) and macrolides (azithromycin and clindamycin) (100%), followed by carbapenem (imipenem) (92.6%) and chloramphenicol (85.2%) (Figure 4). The higher resistance pattern to chloramphenicol aligns with surveillance data from a study assessing antibiotic profiles in bovine *Salmonella* isolates across the U.S. from 2013 to 2022 [107]. Our results support that intensive antibiotic use is a critical driver of high MAR indices. Previous studies revealed that regions with less regulated antibiotic practices frequently report similar resistance patterns, underscoring the direct impact of antimicrobial usage on resistance development [2]. In response to these findings, the FDA’s new rule (GFI 263) will require that farmers and livestock owners obtain a prescription from a licensed veterinarian [108]. This observation may reflect the therapeutic use of these antibiotics for the treatment of *Salmonella* infections among adult cattle. Notably, *S.* Dublin strains in this study exhibited higher resistance profiles, consistent with previous reports. In the U.S., *S.* Dublin has emerged as one of the most MDR serotypes, often associated with resistance to critically important antimicrobial agents [109,110].

To validate the phenotypic resistance patterns, the presence of corresponding ARGs was assessed through targeted PCR analysis. We tested for the presence of key ARGs associated with resistance to β-lactams, aminoglycosides, macrolides, tetracyclines, sulfonamides, and phenicols, providing insights into the genetic basis of the observed resistance patterns. We identified resistance genes for tetracycline (*tetB*) and sulfonamides (sul2), indicating a broad resistance profile among the isolates [85]. Moreover, our isolates harbored several ARGs, including genes resistant to beta-lactamases, including *bla_TEM_*_-1B_*, bla_CMY,_ bla_CTXM_,* and *bla_SHV_*. The *bla_TEM_*_-1B_ gene typically confers resistance to penicillin, such as ampicillin. It may contribute to reduced susceptibility to carbapenems, including meropenem, particularly when additional resistance mechanisms, such as porin loss, are present [111]. Although some ARGs exhibited weak or non-significant correlations with phenotypic resistance, the *bla*_CMY_ gene showed strong associations with β-lactam resistance, including significant correlations with amoxicillin–clavulanic acid (*r* = 0.62, *p* < 0.001), ampicillin (*r* = 0.64, *p* < 0.001), and ceftiofur (*r* = 0.54, *p* < 0.001). Similarly, the *bla_TEM_*_-1B_ gene showed significant positive correlations with resistance to meropenem (*r* = 0.37; *p* < 0.05), amoxicillin–clavulanic acid (*r* = 0.62; *p* < 0.001), ampicillin (*r* = 0.64; *p* < 0.001), and ceftiofur (*r* = 0.54; *p* < 0.001) (Figure 8), highlighting its role in mediating resistance across both penicillin and cephalosporin classes. Importantly, all isolates carried the (*aac(6′)-Ib*) gene, which encodes an aminoglycoside-modifying enzyme that confers resistance to a broad spectrum of aminoglycoside antibiotics [112]. Consistent with the genotypic profile, all isolates demonstrated phenotypic resistance to both neomycin and gentamicin [113]. Similarly, 70.1% of the isolates (19 out of 27) harbored the *ermB2* gene, which encodes an rRNA methyltransferase associated with macrolide resistance [114]. Interestingly, all the isolates (100%, *n* = 27) demonstrated MDR to macrolides, specifically azithromycin and clindamycin. Although phenotypic and genotypic correlations provide valuable insights, they may not capture all underlying resistance mechanisms. Whole-genome sequencing (WGS) is necessary to identify undetected β-lactamases, efflux systems, regulatory mutations, and mobile genetic elements that contribute to resistance. Integrating WGS with phenotypic data would provide a more comprehensive understanding of resistance pathways and virulence gene dissemination in *Salmonella* isolates [115]. While this study observed statistical correlations between phenotypic resistance patterns and the presence of specific resistance genes, it is important to recognize that correlation does not imply causation. Some associations may be spurious or non-causal, potentially resulting from genetic linkage, co-selection, or the presence of shared mobile genetic elements rather than direct gene-function relationships [116,117]. This study highlights the critical need for sustained surveillance and genetic characterization of *Salmonella* in cattle. Despite the relatively low prevalence, the detection of a broad array of virulence factors and antimicrobial resistance genes, particularly those associated with biofilm formation, motility, and immune evasion, raises concerns about persistence within herds and the potential for both intraspecies and interspecies transmission. These factors may also serve as potential targets for the development of novel therapeutics and control strategies [118,119].

## 5. Conclusions

Our study highlights the significant concern of AMR in *Salmonella* isolates from cattle, along with their notable virulence potential and biofilm formation capabilities. *Salmonella* Dublin was the most prevalent serotype, along with eleven other serotypes. Swarming and swimming motility were observed in most of the isolates, in addition to biofilm production. Furthermore, our isolates harbored different virulence genes and ARGs. The aminoglycoside resistance gene *aac(6′)-Ib* was detected in all the isolates (100% prevalence), and all the isolates also harbored multiple virulence genes, including *invA*, *hilA*, *fimA*, *csgA*, *csgB*, and *flhD.* Strikingly, all the isolates exhibited an MDR phenotype, characterized by high MAR index values, underscoring their potential threat to both animal and public health. The emergence of MDR *Salmonella* in cattle poses a critical threat to the livestock industry and public health. Thorough investigations using whole-genome sequencing are needed to fully understand the AMR and virulence gene profiles of pathogens. Simultaneously, studying transmission dynamics is crucial for tracing the sources of *Salmonella* infections in cattle and understanding how these infections can spread to the environment. Additionally, elucidating the distribution of ARGs and virulence factors offers valuable insights for identifying potential drug targets and guiding the development of more effective antibiotic alternatives. These efforts will assist veterinary services and regulatory authorities in implementing effective biosecurity measures to prevent the further dissemination of these pathogens. These findings underscore the importance of implementing integrated One Health–based monitoring strategies to mitigate environmental dissemination and protect public and animal health.

## Figures and Tables

**Table 1:** Occurrence of *Salmonella* isolates categorized by gender, age group, organ of isolation, and serotypes.

Group	Variable	Count (*n*)	Percentage (%)	95% CI (Lower–Upper)	*p*-Value
Gender	Female	16	59.3	40.7–77.8	0.4421
	Male	11	40.7	22.2–59.3	0.4421
Age Group	Neonatal	3	11.1	0.0–23.0	<0.05
	Calf	5	18.5	3.9–33.2	<0.05
	Juvenile	9	33.3	15.6–51.1	<0.05
	Adult	10	37	18.8–55.3	<0.05
Organ	Intestine	21	77.8	62.1–93.5	<0.0001
	Liver	2	7.4	0.0–17.3	<0.05
	Colon	2	7.4	0.0–17.3	<0.05
	Lung	1	3.7	0.0–10.8	<0.05
	Kidney	1	3.7	0.0–10.8	<0.05
Serotypes	Dublin	8	29.6	12.4–46.9	<0.05
	Typhimurium	3	11.1	0.0–23.0	<0.05
	Muenster	3	11.1	0.0–23.0	<0.05
	Montevideo	2	7.4	0.0–17.3	<0.05
	Thompson	2	7.4	0.0–17.3	<0.05
	Worthington	2	7.4	0.0–17.3	<0.05
	Anatum	2	7.4	0.0–17.3	<0.05
	Cerro	1	3.7	0.0–10.8	<0.05
	Hartford	1	3.7	0.0–10.8	<0.05
	Meleagridis	1	3.7	0.0–10.8	<0.05
	Newport	1	3.7	0.0–10.8	<0.05
	III 38:(k): z35	1	3.7	0.0–10.8	<0.05

Percentages are presented with 95% confidence intervals, calculated using the Clopper–Pearson method, based on a total of 27 *Salmonella* isolates. *Chi*-square or Fisher’s exact test was used for group comparisons, depending on the expected cell counts.

## Data Availability

The original contributions presented in this study are included in the article/supplementary material. Further inquiries can be directed to the corresponding author(s).

## Supplementary Materials

The following supporting information can be downloaded at: https://www.mdpi.com/article/10.3390/antibiotics14070689/s1, Table S1. Breakpoints and antibiotic concentrations for *Salmonella* spp. according to CLSI M100 and CLSI VET01S guidelines. Table S2. Oligonucleotide primer sequences and their corresponding virulence genes. Table S3. Oligonucleotide primer sequences and their corresponding Antimicrobial Resistant Genes (ARGs). Table S4. Distribution of isolates by response animal species, gender, variable age, site of isolation, and *Salmonella* serotype. Table S5. Biofilm formation of *Salmonella* isolates. Table S6. Resistance frequencies for each class. Table S7. Multidrug Resistance Profile of *Salmonella* Isolates. Figure S1 (A) swimming motility and (B) Swarming motility for C26, C19, C17, and C16 on semi-solid agar plates after 12 h. of incubation at 37 °C, in comparison to *S.* Typhimurium ATCC 14028, *P. mirabilis* ATCC 35659, and *Rhodococcus equi*. References [120,121,122,123,124,125,126,127,128,129,130,131,132,133,134,135,136,137,138,139,140] are cited in the supplementary materials.
