# Supplementary material for: Multidrug Resistance and Virulence Traits of Salmonella enterica Isolated from Cattle: Genotypic and Phenotypic Insights
Source: Antibiotics (Basel). 2025 Jul 8;14(7):689. doi: 10.3390/antibiotics14070689 (PMC12291950; doi:10.3390/antibiotics14070689)
Supplement: Supplementary file 1 [file antibiotics-14-00689-s001.zip › antibiotics-3714000-supplementary.pdf]

Table S1. Breakpoints and antibiotic concentrations for *Salmonella* spp. according to CLSI M100 & CLSI VET01S guidelines.

Class	Antibiotic	Abbreviation	Concentration (μg/mL)	Reference
Aminoglycoside	Neomycin	NEO	0.12-2	CLSI VET01S
	Gentamicin	GEN	1-8	CLSI VET01S
	Amikacin	AMK	4-32	CLSI M100
Penicillin (β-lactam)	Amoxicillin & Clavulanic Acid	AMC	0.25/0.12-8/4	CLSI M100
	Ampicillin	AMP	0.25-8	CLSI M100
Carbapenem	Meropenem	MEM	0.5-64	CLSI M100
	Imipenem	IPM	1-8	CLSI M100
Cephalosporin (β-lactam)	Ceftazidime	CAZ	0.5-64	CLSI M100
	Ceftiofur	CEF	0.25-8	CLSI VET01S
Tetracyclines	Tetracycline	TET	2-8	CLSI VET01S
	Doxycycline	DOX	2-16	CLSI M100
Sulfonamide	Trimethoprim & Sulfamethoxazole	SXT	0.5/9.5-4/76	CLSI M100
Fluoroquinolone	Levofloxacin	LVX	0.5-64	CLSI M100
	Ciprofloxacin	CIP	0.5-64	CLSI M100
	Enrofloxacin	ENR	0.12-2	CLSI VET01S
Phenicols	Florfenicol	FLO	0.25-8	CLSI VET01S
	Chloramphenicol	CHL	4-32	CLSI M100
Macrolide	Azithromycin	AZM	0.5-64	CLSI M100
	Clindamycin	CLI	0.5-4	CLSI VET01S

Table S2. Oligonucleotide primer sequences and their corresponding virulence genes.

Function	Gene	Sequence	Annealing Temperature (°C)	Amplicon Size	Reference
Adhesion & Attachment	*fimA*	F-AACGGAGCCGACAGGATGCCGAAACCGGG<br>R-CTGCCAGGACCGGTAAACGCATTCGTGCGG	64	881	This study
	fimD	F-AACGGAGCCGACAGGATGCCGAAACCGGG<br>R-CTGCCAGGACCGGTAAACGCATTCGTGCGG	58	125	This study
Cell Invasion & Intracellular Survival	*invA*	F-CTCGCCTTTGCTCCTTTTAG<br>R-GCCATGGTATGGATTTGTCC	58	211	[1]
	hilA	F-TTAAACATGTCGCCCAAACAGC<br>R- GCAAACTCCCGATGTAT	55	216	[2]
	intA	F-CCTCCCGCACGATGATC<br>R-TCCACGCATCGTCAGGC	52	280	[3]
	intB	F- TTATTGCTGGGATTAGGC<br>R- ACGGCTACCCTCTGTTATC	63	250	[4]
	spiA	F-CCAGGGTCGTTAGTGTATTAGTGTATTGCGTGAGATG<br>R-CGCGTAACAAAGAACCCGTAGTAGTGATGGATT	67	102	This study
T3SS	*spi4D*	F- ATGCTTAATATTCAAAATTATTCCG<br>R- TCCTTGCAGGAAGCTTTTG	52	1129	[5]
	sopB	F-GCTCTAGACCTCAAGACTCAAGATG<br>R-GCGGCCGCTACGCAGGAGTAAATCGGTG	55	1987	[5]
Host Immune Suppressor	*spvC*	F-ACTCCTTGCACAACCAAATGCGGA<br>R-TGTCTTCTGCATTTCGCCACC	53	571	[6]
	csgA	F- GCAATCGTATTCTCCGGTAG<br>R- GATGAGCGGTCGCGTTGTTA	53	418	[7]
Biofilm	*csgB*	F: ATCAGGCGGCCATTATTGGTCAAG<br>R: TGCTGTTTTCTGCGTACCGTACTG	61	275	[8]
	siiC	F- AGCTACAGCAACTCTCATTGATTT<br>R-CCCTGACCATGAACCACTGAA	56	420	[9]
T1SS	*siiA*	F-ACGACTGGGATATGAACGGGGAA<br>R- TCGTTGTACTTGATGCTGCGGAG	60	107	[10]
	fljB	F-TGGATGTATCGGGTCTTGATG<br>R-CACCAGTAAAGCCACCAATAG	52	200	[11]
	fliC	F-ACTGCTAAAACCACTACT<br>R-TGGAGACTTCGGTTGCGTAG	48	133	[12]

Motility	*flhD*	F-CGTTTGATCGTCCAGGACAA<br>R-TGTTTGCCATCTCTTCGTTGAT	57	190	[13]

Table S3. Oligonucleotide primer sequences and their corresponding Antimicrobial Resistant Genes (ARGs).

Function	Gene	Sequence	Annealing Temperature (°C)	Amplicon Size	Reference
β-lactamase	bla_{TEM-1B}	F-ATGAGTATTCAACATTTCCG<br>R-ACCAATGCTTAATCAGTGAG	55	964	[14]
	bla_{CMY}	F-TGGCCGTTGCCGTTATCTAC<br>R-CCCGTTTTATGCACCCATGA	63	870	[15]
	bla_{OXA-9}	F-ACCAGATTCAACTTTCAA<br>R-TCTTGGCTTTTATGCTTG	53	590	[14]
	bla_{SHV2}	F-TTCGCCTGTGTATTATCTCCCTG<br>R-TTAGCGTTGCCAGTGCTCG	53	854	[14]
	bla_{CTX-M}	F-GAGTTTCCCCATTCCGTTTC<br>R-CAGAATAAGGAATCCCATGGT	55	909	[14]
Aminoglycoside	*aac(3) -Iva*	F-ATTGAAGATTTGCCAGAACA<br>R-CACTATCATAACCACTACCG	56.5	178	[16]
	aac(6′) -Ib	F-TTGCGATGCTCTATGAGTGGCTA<br>R-CTCGAATGCCTGGCGTGTTT	58	482	[15]
	aadB	F-GGCAGACGAAGCGTATGAA<br>R-CGACCTGAAAGCGGCAC	56	244	[15]
Phenicol	*floR*	F-ATCCAACTCACGTTGAGCC<br>R-TTGGATGCAGAAGTAGAACG	52	868	[14]
	catB	F-CGGATTCAGCCTGACCACC<br>R-ATACGCGGACACCTTCCTG	63	461	[17]
Sulfonamide	*sul2*	F-GCGCTCAAGGCAGATGGCATT<br>R-GCGTTTGATACCGGCACCCGT	55	285	[14]
Streptomycin	*strA*	F-CCAATCGCAGATAGAAGGC<br>R-CTTGGTGATAACGGCAATTC	55	548	[16]
Tetracycline	*tetB*	F-GAGACGCAATCGAATTCGG<br>R-TTTAGTGGCTATTCTTCCTGCC	52	228	[14]
Colistin	*mcr1*	F-AGTCCGTTTGTTCTTGTGGC<br>R-AGATCCTTGGTCTCGGCTTG	58	320	[18]
	mcr2	F-CAAGTGTGTTGGTCGCAGTT<br>R-TCTAGCCGACAAGCATACC	60	715	[18]
	mcr3	F-AAATAAAAATTGTTCCGCTTATG<br>R- AATGGAGATCCCCGTTTTT	58	929	[18]
	mcr4	F- TCACTTTCATCACTGCGTTG<br>R-TTGGTCCATGACTACCAATG	58	1116	[18]

	mcr5	F-ATGCGGTTGTCTGCATTTATC<br>R-TCATTGTGGTTGTCCTTTTCTG	50	1644	[19]
	mcr6	F-AGCTATGTCAATCCCGTGAT<br>R- ATTGGCTAGGTTGTCAATC	55	252	[20]
	mcr7	F-GCCCTTCTTTTCGTTGTT<br>R-GGTTGGTCTCTTTCTCGT	55	551	[20]
	mcr8	F-TCAACAATTCTACAAAGCGTG<br>R-AATGCTGCGCGAATGAAG	55	856	[20]
	mcr9	F-TTCCCTTTGTTCTGGTTG<br>R-GCAGGTAATAAGTCGGTC	55	1011	[20]
Macrolides	*ermB2*	F-GAAAAGGTACTCAACCAAATA<br>R-GTAACGGTACTTAAATTGTTTAC	52	639	[21]

Table S4. Distribution of isolates by response animal species, gender, variable age, site of isolation, and *Salmonella* serotype.

Isolates	Animal Species	Gender	Age Class	Site of Isolation	*Salmonella* Serotype	Date
C1	Cattle	Female	Juvenile	Intestine	Typhimurium	January-22
C2	Cattle	Female	Adult	Intestine	III 38:(k):z35	February-22
C3	Cattle	Female	Adult	Intestine	Typhimurium	March-22
C4	Cattle	Male	Calf	Intestine	Dublin	March-22
C5	Cattle	Female	Juvenile	Liver	Dublin	June-22
C6	Cattle	Female	Adult	Intestine	Cerro	September-22
C7	Cattle	Male	Juvenile	Intestine	Muenster	September-22
C8	Cattle	Female	Adult	Intestine	Thompson	October-22
C9	Cattle	Female	Adult	Intestine	Typhimurium	October-22
C10	Cattle	Male	Calf	Intestine	Hartford	October-22
C11	Cattle	Male	Juvenile	Intestine	Dublin	November-22
C12	Cattle	Female	Calf	Intestine	Dublin	November-22
C13	Cattle	Female	Juvenile	Intestine	Dublin	November-22
C14	Cattle	Female	Calf	liver	Dublin	December-22
C15	Bovine	Male	Calf	Lung	Worthington	March-23
C16	Cattle	Male	Juvenile	Intestine	Anatum	December-23
C17	Bovine	Female	Adult	Intestine	Meleagridis	February-23
C18	Bovine	Male	Neonatal	Intestine	Muenster	February-23
C19	Bovine	Female	Adult	Intestine	Worthington	March-23
C20	Bovine	Female	Neonatal	Kidney	Dublin	April-23
C21	Bovine	Male	Neonatal	Intestine	Dublin	April-23
C22	Bovine	Male	Juvenile	Colon	Montevideo	October-23
C23	Bovine	Female	Adult	Colon	Anatum	October-23
C24	Cattle	Female	Juvenile	Intestine	Muenster	October-23
C25	Cattle	Male	Juvenile	Intestine	Newport	November-23
C26	Cattle	Male	Adult	Intestine	Montevideo	November-23
C27	Cattle	Female	Adult	Intestine	Dublin	November-23

Table S5. Biofilm formation of *Salmonella* isolates.

Salmonella Isolates	Mean (OD_{550})	STDV	Biofilm Category
C1	0.1520	0.024	MBP
C2	0.2193	0.032	SBP
C3	0.1333	0.021	WBP
C4	0.1767	0.058	MBP
C5	0.1698	0.023	MBP
C6	0.1910	0.043	MBP
C7	0.2035	0.021	SBP
C8	0.2438	0.053	SBP
C9	0.2228	0.025	SBP
C10	0.1595	0.018	MBP
C11	0.1972	0.028	MBP
C12	0.1463	0.009	MBP
C13	0.1798	0.022	MBP
C14	0.2885	0.063	SBP
C15	0.2053	0.067	SBP
C16	0.2235	0.051	SBP
C17	0.2978	0.048	SBP
C18	0.2370	0.019	SBP
C19	0.1682	0.041	MBP
C20	0.1278	0.036	MBP
C21	0.1260	0.041	MBP
C22	0.2220	0.04	SBP
C23	0.4087	0.054	MBP
C24	0.1362	0.012	WBP
C25	0.2288	0.092	SBP
C26	0.2185	0.037	SBP
C27	0.2233	0.057	SBP

Table S6. Resistance frequencies for each class.

Class	Antibiotic	Count (n)	Frequency (%)
Aminoglycoside	NEO	27	100
	GEN	27	100
	AMK	8	29.6
Penicillin (β-lactam)	AMC	1	3.7
	AMP	8	29.6
Carbapenem (β-lactam)	MEM	1	3.7
	IPM	25	92.6
Cephalosporin (β-lactam)	CAZ	4	14.8
	CEF	7	25.9
Tetracyclines	TET	14	51.9
	DOX	5	18.5
Fluoroquinolone	SXT	9	33.3
	LVX	10	37.0
	CIP	5	18.5
Sulfonamide	ENR	3	11.1
Phenicols	FLO	0	0
	CHL	23	85.2
Macrolide	AZM	27	100
	CLI	27	100

Table S7. Multidrug Resistance Profile of *Salmonella* Isolates.

Isolates	*Salmonella* Serotypes	Resistance Profile	MDR Classes	MAR Index
C1	Typhimurium	NEO, GEN, LEV, CIP, CHL, AZM, CLI	Aminoglycoside, Fluoroquinolone, Phenicols, Macrolides	0.37
C2	III 38:(k): z35	NEO, GEN, CAZ, IPM, LVX, CIP, CHL, AZM, CLI	Aminoglycoside, Fluoroquinolone, Carbapenem, Macrolides, Phenicols, Cephalosporin	0.47
C3	Typhimurium	NEO, GEN, IPM, LVX, CIP, CHL, AZM, CLI	Aminoglycoside, Carbapenem, Fluoroquinolone, Chloramphenicols, Macrolides	0.42
C4	Dublin	NEO, GEN, AMK, AMP, CEF, IPM, TET, CIP, CHL, AZM, CLI	Aminoglycoside, Penicillin, Carbapenem, Cephalosporin, Tetracyclines, Fluoroquinolone, Phenicols, Macrolides	0.58
C5	Dublin	NEO, GEN, AMK, AMP, CEF, IPM, TET, LVX, CIP, CHL, AZM, CLI	Aminoglycoside, Penicillin, Carbapenem, Cephalosporin, tetracyclines, Fluoroquinolone, Phenicols, Macrolides	0.63
C6	Cerro	NEO, GEN, AMP, CEF, IPM, TET, ENR, CHL, AZM, CLI	Aminoglycoside, Penicillin, Carbapenem, Cephalosporin, tetracyclines, Fluoroquinolone, Phenicols, Macrolides	0.53
C7	Muenster	NEO, GEN, IPM, CHL, AZM, CLI	Aminoglycoside, Carbapenem, Phenicols, Macrolides	0.32
C8	Thompson	NEO, GEN, IPM, CHL, AZM, CLI	Aminoglycoside, Carbapenem, Phenicols, Macrolides	0.32
C9	Typhimurium	NEO, GEN, IPM, CHL, AZM, CLI	Aminoglycoside, Carbapenem, Phenicols, Macrolides	0.32
C10	Hartford	NEO, GEN, IPM, LVX, CHL, AZM, CLI	Aminoglycoside, Carbapenem, Fluoroquinolone, Phenicols, Macrolides	0.37
C11	Dublin	NEO, GEN, AMK, AMP, CEF, IPM, TET, LVX, CIP, ENR, CHL, AZM, CLI	Aminoglycoside, Penicillin, Carbapenem, Cephalosporin, tetracyclines, Fluoroquinolone, Phenicols, Macrolides	0.68
C12	Dublin	NEO, GEN, AMK, AMP, CEF, IPM, TET, LVX, CIP, ENR, CHL, AZM, CLI	Aminoglycoside, Penicillin, Carbapenem, Cephalosporin, Tetracyclines, Fluoroquinolone, Phenicols, Macrolides	0.68

C13	Dublin	NEO, GEN, IPM, TET, CHL, AZM, CLI	Aminoglycoside, Carbapenem, Tetracyclines, phenicols, Macrolides	0.37
C14	Dublin	NEO, GEN, AMK, AMP, CEF, MEM, IPM, TET, LVX, CIP, ENR, CHL, AZM, CLI	Aminoglycoside, Penicillin, Carbapenem, Cephalosporin, Tetracyclines, Fluoroquinolone, Phenicols, Macrolides	0.74
C15	Worthington	NEO, GEN, CAZ, IPM, TET, CIP, SXT, CHL, AZM, CLI	Aminoglycoside, Carbapenem, Cephalosporin, Tetracyclines, Fluoroquinolone, Sulfonamide, Phenicols, Macrolides	0.53
C16	Anatum	NEO, GEN, AMP, CEF, TET, DOX, CHL, AZM, CLI	Aminoglycoside, Penicillin, Cephalosporin, Tetracyclines, Phenicols, Macrolides	0.47
C17	Meleagridis	NEO, GEN, IPM, TET, CHL, AZM, CLI	Aminoglycoside, Carbapenem, tetracyclines, Sulfonamide, Phenicols, Macrolides	0.37
C18	Muenster	NEO, GEN, IPM, TET, SXT, CHL, AZM, CLI	Aminoglycoside, Carbapenem, tetracyclines, phenicols, Macrolides	0.42
C19	Worthington	NEO, GEN, CAZ, IPM, TET, SXT, CHL, AZM, CLI	Aminoglycoside, Carbapenem, Cephalosporin, tetracyclines, Sulfonamide, phenicols, Macrolides	0.47
C20	Thompson	NEO, GEN, IPM, LVX, CIP, CHL, AZM, CLI	Aminoglycoside, Carbapenem, Fluoroquinolone, Tetracyclines, Sulfonamide, phenicols, Macrolides	0.42
C21	Dublin	NEO, GEN, IPM, CHL, AZM, CLI	Aminoglycoside, Carbapenem, phenicols, Macrolides	0.32
C22	Montevideo	NEO, GEN, IPM, TET, CHL, AZM, CLI	Aminoglycoside, Carbapenem, tetracyclines, phenicols, Macrolides	0.37
C23	Anatum	NEO, GEN, IPM, CHL, AZM, CLI	Aminoglycoside, Carbapenem, Phenicols, Macrolides	0.32
C24	Muenster	NEO, GEN, AMK, IPM, TET, DOX, AZM, CLI	Aminoglycoside, Carbapenem, Tetracyclines, Macrolides	0.42
C25	Newport	NEO, GEN, AMK, IPM, DOX, AZM, CLI	Aminoglycoside, Carbapenem, Tetracyclines, Macrolides	0.37
C26	Montevideo	NEO, GEN, AMK, IPM, TET, DOX, AZM, CLI	Aminoglycoside, Carbapenem, Tetracyclines, Macrolides	0.42

C27	Dublin	NEO, GEN, AMC, AMP, CAZ, MEM, IPM, TET, DOX, ENR, AZM, CLI	Aminoglycoside, Penicillin, Cephalosporin, Carbapenem, Tetracyclines, Fluoroquinolone, Macrolides	0.58

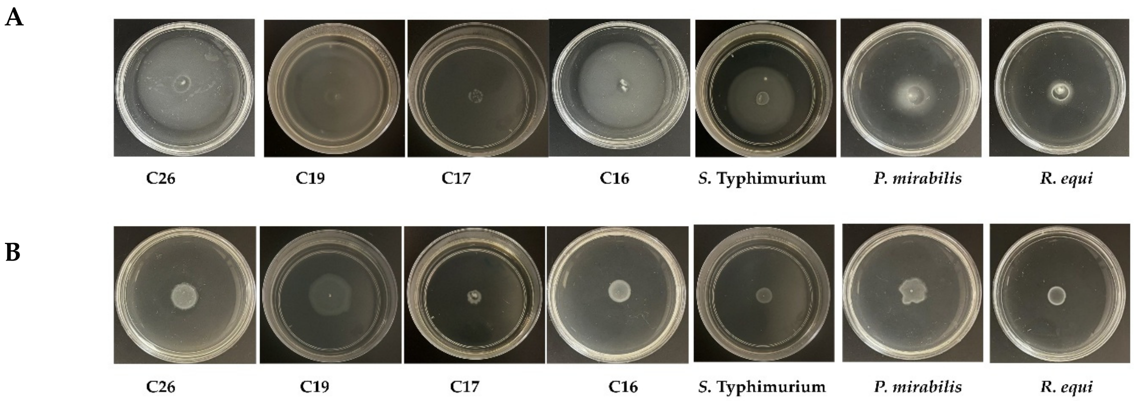

Figure S1 (A) swimming motility & (B) Swarming motility for C26, C19, C17, and C16 on semi-solid agar plates after 12 h. of incubation at 37 °C, in comparison to *S.* Typhimurium ATCC 14028, *P. mirabilis* ATCC 35659, and *Rhodococcus equi.*

References

1. Fazl, A.A., et al., *Molecular detection of invA, ssaP, sseC and pipB genes in Salmonella Typhimurium isolated from human and poultry in Iran.* Afr J Microbiol ReS, 2013. **7**(13): p. 1104-8.
2. Li, G., et al., *Punicalagin inhibits Salmonella virulence factors and has anti-quorum-sensing potential.* Applied and environmental microbiology, 2014. **80**(19): p. 6204-6211.
3. Bass, L., et al., *Incidence and characterization of integrons, genetic elements mediating multiple-drug resistance, in avian Escherichia coli.* Antimicrobial Agents and Chemotherapy, 1999. **43**(12): p. 2925-2929.
4. Goldstein, C., et al., *Incidence of class 1 and 2 integrases in clinical and commensal bacteria from livestock, companion animals, and exotics.* Antimicrobial agents and chemotherapy, 2001. **45**(3): p. 723-726.
5. Sever, N.K. and M. Akan, *Molecular analysis of virulence genes of Salmonella Infantis isolated from chickens and turkeys.* Microbial pathogenesis, 2019. **126**: p. 199-204.
6. Turki, Y., et al., *Molecular typing, antibiotic resistance, virulence gene and biofilm formation of different Salmonella enterica serotypes.* The Journal of General and Applied Microbiology, 2014. **60**(4): p. 123-130.
7. Allen, S.E., et al., *Antimicrobial resistance in generic Escherichia coli isolates from wild small mammals living in swine farm, residential, landfill, and natural environments in southern Ontario, Canada.* Applied and environmental microbiology, 2011. **77**(3): p. 882-888.
8. Lin, L., et al., *Inhibitory effect of cold nitrogen plasma on Salmonella Typhimurium biofilm and its application on poultry egg preservation.* Lwt, 2020. **126**: p. 109340.
9. Xu, Y., et al., *The role of egg yolk in modulating the virulence of Salmonella enterica serovar Enteritidis.* Frontiers in Cellular and Infection Microbiology, 2022. **12**: p. 903979.
10. Hassena, A.B., et al., *Real time PCR gene profiling and detection of Salmonella using a novel target: the siiA gene.* Journal of microbiological methods, 2015. **109**: p. 9-15.
11. Bearson, B.L. and S.M. Bearson, *The role of the QseC quorum-sensing sensor kinase in colonization and norepinephrine-enhanced motility of Salmonella enterica serovar Typhimurium.* Microbial pathogenesis, 2008. **44**(4): p. 271-278.
12. Ganesan, V., et al., *Detection of Salmonella in Blood by PCR using iroB gene.* Journal of clinical and diagnostic research: JCDR, 2014. **8**(11): p. DC01.
13. Shah, T., et al., *Trans-cinnamaldehyde nanoemulsion reduces Salmonella Enteritidis biofilm on steel and plastic surfaces and downregulates expression of biofilm associated genes.* Poultry Science, 2025. **104**(5): p. 105086.
14. Chen, Z., et al., *Prevalence, antimicrobial resistance, virulence genes and genetic diversity of Salmonella isolated from retail duck meat in southern China.* Microorganisms, 2020. **8**(3): p. 444.
15. Cao, Z., et al., *Prevalence and antimicrobial resistance of Salmonella isolates from goose farms in Northeast China.* Iranian Journal of Veterinary Research, 2020. **21**(4): p. 287.

16. Asgharpour, F., et al., *Molecular detection of class 1, 2 and 3 integrons and some antimicrobial resistance genes in Salmonella Infantis isolates.* Iranian Journal of Microbiology, 2018. **10**(2): p. 104.
17. Chuanchuen, R. and P. Padungtod, *Antimicrobial resistance genes in Salmonella enterica isolates from poultry and swine in Thailand.* Journal of Veterinary Medical Science, 2009. **71**(10): p. 1349-1355.
18. Rebelo, A.R., et al., *Multiplex PCR for detection of plasmid-mediated colistin resistance determinants, mcr-1, mcr-2, mcr-3, mcr-4 and mcr-5 for surveillance purposes.* Eurosurveillance, 2018. **23**(6): p. 17-00672.
19. Borowiak, M., et al., *Identification of a novel transposon-associated phosphoethanolamine transferase gene, mcr-5, conferring colistin resistance in d-tartrate fermenting Salmonella enterica subsp. enterica serovar Paratyphi B.* Journal of Antimicrobial Chemotherapy, 2017. **72**(12): p. 3317-3324.
20. Borowiak, M., et al., *Development of a novel mcr-6 to mcr-9 multiplex PCR and assessment of mcr-1 to mcr-9 occurrence in colistin-resistant Salmonella enterica isolates from environment, feed, animals and food (2011–2018) in Germany.* Frontiers in microbiology, 2020. **11**: p. 80.
21. Gan, T., et al., *Antimicrobial resistance and genotyping of Staphylococcus aureus obtained from food animals in Sichuan Province, China.* BMC veterinary research, 2021. **17**(1): p. 177.
